# Therapeutic potential of HDAC6 in amyotrophic lateral sclerosis

**DOI:** 10.15698/cst2018.01.120

**Published:** 2017-12-19

**Authors:** Wenting Guo, Ludo Van Den Bosch

**Affiliations:** 1 KU Leuven-Department of Neurosciences, Experimental Neurology, Leuven, Belgium.; 2 VIB, Center for Brain & Disease Research, Laboratory of Neurobiology, Leuven, Belgium.

**Keywords:** Histone deacetylase 6, motor neuron, neurodegeneration, acetylation, axonal transport, therapy, mitochondria

## Abstract

Amyotrophic lateral sclerosis (ALS) is the most common fatal motor neuron disease in adults and no effective treatments exist. Mutations in *FUS* are one of the genetic causes of familial ALS. We used induced pluripotent stem cells (iPSCs) derived from FUS-ALS patients to investigate the pathological mechanism. We observed hypo-excitability, cytoplasmic FUS localization and axonal transport defects of different cargoes in motor neurons differentiated from these iPSCs. Pharmacological inhibition and genetic silencing of histone deacetylase 6 (HDAC6) restored the axonal transport defects. Moreover, the disturbed association between mitochondria and the endoplasmic reticulum (ER) was also reversed by inhibition of HDAC6. The positive effects of HDAC6 inhibition were linked to an increase in the acetylation level of α-tubulin, one of the building blocks of the microtubules. In conclusion, HDAC6 inhibition could be a potential new therapeutic strategy for ALS.

Amyotrophic lateral sclerosis (ALS) is a neurodegenerative disorder characterized by the selective death of motor neurons in the motor cortex, brainstem and spinal cord. This leads to progressive muscle weakness, paralysis and ultimately the death of the patient on average 2-5 years after the diagnosis. While ALS is a sporadic disease in 90% of cases, 10% of patients are classified as familial ALS. The most common genetic causes of familial ALS are mutations in *SOD1*, *TARDBP* and *FUS, *as well as hexanucleotide repeats in *C9ORF72*. There is currently no cure for ALS.

We focused in our study on ALS induced by mutations in *FUS*. This gene encodes a DNA/RNA binding protein with a multitude of functions related to DNA and RNA. Although FUS mainly localizes into the nucleus, it also shuttles between the nucleus and the cytoplasm. Under stress conditions, FUS localizes to stress granules (SGs) which are cytoplasmic messenger ribonucleoprotein (mRNP) particles that are formed after stress. At a certain moment, this can interfere with other physiological processes and this can result in cell death.

We started from fibroblasts of patients with two different mutations in FUS and controls. We created induced pluripotent stem cells (iPSCs) using non-integrating Sendai virus expressing the four classical dedifferentiation factors. The advantage of this strategy is that the mutated gene is not overexpressed and it is present in the genetic background of the patient. We characterized several iPSC lines and have proven that these lines can give rise to the different germ layers. We also adapted a protocol to differentiate these iPSCs into highly pure motor neurons. Based on the observation that all iPSC lines have a similar efficiency to differentiate into motor neurons, we conclude that mutation in FUS have no effect on the differentiation capacity of these cells. This could be due to the fact that this differentiation process resembles what happens during embryonic development. However, we observed hypo-excitability, cytoplasmic FUS mislocalization and progressive axonal transport defects in the cells derived from ALS patients.

Physiological changes were demonstrated by several other studies in different iPSC-derived ALS models. Both hypo-excitability and hyper-excitability were reported. In our study, we observed patient derived motor neurons presented with a reduced frequency of (action potential) APs and postsynaptic currents at week 7 of differentiation. Therefore, hypo-excitability was confirmed in our patient-derived motor neurons. Hypo-excitability was confirmed in our patient-derived motor neurons. Although hypo-excitation which is supposed to mimic the aging stage of a neuron could be a late phenotype of ALS. We didn’t observe obvious cell death in the motor neurons derived from patient iPSCs. Thus, at this stage we consider the hypo-excitability as an early pathological change rather than a late stage phenotype.

Cytoplasmic FUS localization was observed in all cell types, including fibroblasts, iPSCs and motor neurons. This suggests that this pathological change could serve as a potential hazard, but that it is not sufficient to cause motor neuron death in ALS. We didn’t observe obvious cell death in our motor neuron cultures. We believe that this is an early phenotype in ALS and that it could become more severe with aging or under stress. Cytoplasmic FUS localization and axonal transport defects were rescued in genetically corrected isogenic iPSC-derived motor neurons. As a consequence, it seems that the mutation itself is sufficient to induce these phenotypes. Moreover, the same phenotypes were detected in motor neurons generated from embryonic stem cells in which two different mutant FUS genes were overexpressed from the AAVS1 locus, while overexpression of wild type FUS had no obvious effect. In addition, knock down of FUS did not induce these phenotypes. As a consequence, our data are in line with the hypothesis that a ‘gain of function’ of mutant FUS induces different ALS-related phenotypes. FUS can bind and regulate mRNAs of several motor proteins including KIF5C, KIF1B and KIF3A153. All these motor proteins are involved in axonal transport of mitochondria and vesicles. KIF5C belongs to the Kinesin1 family which drives anterograde transport of mitochondria along axons. Deficiency of anterograde transport will eventually results in energy deprivation and decreased levels of neurotransmitter at the presynaptic terminal. We assume that if cytoplasmic mutant FUS starts binding these accumulated proteins, more SGs may form thereafter. In another way around, the initial accumulation of mutant FUS along the axon will form aggregates which recruit SGs under cell stress. Since mutant FUS are not dissolvable, SGs accumulate along the axon, which will worsen the axonal transport efficiency and speed up the "dying back" process.

Axonal transport of mitochondria and other organelles or proteins is particularly important in the long axons of motor neurons, which extend a great distance from the soma. The longest and largest axon with the highest metabolic demand seem to be the most susceptible to the "dying back" phenomenon which can provide an explanation for the selective motor neuron vulnerability seen in ALS. In order to rescue axonal transport defects, we used antisense oligonucleotides (ASOs) to knock down HDAC6 at the mRNA level with almost half. We observed that this resulted in the restoration of axonal transport defects in ALS patient derived motor neurons. As a consequence, we obtained additional evidence that HDAC6 could play an important role in ALS. Moreover, we could also rescue both mitochondrial and ER vesicle transport defects by using two different HDAC6 inhibitors. HDAC6 inhibition also increased the total number of ER vesicles. The question arises which underlying mechanism(s) is responsible for this rescue of axonal transport defects by HDAC6 inhibition. We observed a significant increase of the acetylation level of α-tubulin in motor neurons after treating with HDAC6 inhibitors as α-tubulin is the major substrate of HDAC6. Moreover, we also detected a slight decrease of the acetylation level of α-tubulin in patient-derived motor neurons compared to their isogenic controls. This emphasizes the importance of the acetylation level of α-tubulin in maintaining the efficiency of axonal transport. Similar observations were reported in other neurodegenerative disease models. Correction of the α-tubulin acetylation level by HDAC6 inhibition increased axonal transport *in vitro* and was also associated with a rescue of the phenotype. We did not observe an effect of HDAC6 inhibition on the cytoplasmic FUS localization in fibroblasts from ALS patients. The therapeutic effect of HDAC6 inhibitors seems to be mainly related to a direct effect on the increase of the acetylation level of α-tubulin in the microtubules, rather than to an indirect effect on the intracellular localization of mutant FUS (**Figure 1**). In addition, we observed a rescue of the disturbed mitochondria and ER overlay combined with an upregulation of phosphatidylcholine (PC) level in patient-derived motor neurons after treatment with the HDAC6 inhibitors. Mitochondria-associated ER membranes (MAMs) facilitate phospholipid exchange in between and the PC level is a marker for MAMs. MAMs prefer localize along acetylated microtubules. Therefore, we assume that HDAC6 inhibition rescue the overlay of mitochondria and ER by providing more localization spot for MAMs (**Figure 1**).

**Figure 1:**
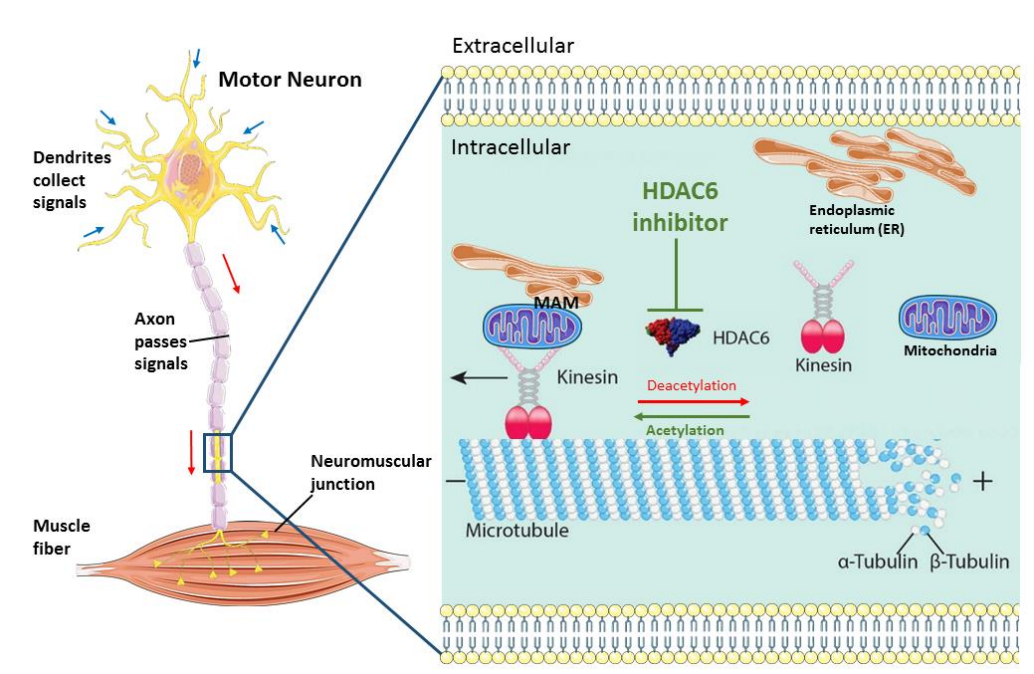
FIGURE 1: Schematic overview of the machinery of HDAC6 inhibition in rescuing axonal transport defects in ALS. Axonal transport cargoes, such as ER and mitochondria, anchored to the motor protein kinesin. The molecular motors kinesin travel along the microtubules with their bound cargoes in an anterograde direction. HDAC6 is an important regulator of axonal transport by regulating the acetylation state of α-tubulins which build up microtubules with β-tubulins. In stabilized microtubules, the minus end facing the soma and dynamic plus end facing the synaptic terminals. Under cell stress, the motor neuron start ‘dying back’ from the distal part of axon caused by insufficient metabolic supply. HDAC6 inhibition increase the acetylation level of microtubules and increase the localization of MAMs along microtubules and finally speed up axonal transport of ER and mitochondria. (This image is constructed based on the images from Servier Medical Art (http://smart.servier.com and Neurobiol Dis. 2017 Sep; 105:300-320.doi: 10.1016/j.nbd.2017.02.009).

The FDA-approved drugs for ALS at this moment are riluzole and edaravone. Although the exact mechanism responsible for the therapeutic effect is not known, riluzole has clear anti-excitotoxic properties. It prevents glutamate release from presynaptic terminals and it can also block glutamate receptors. The therapeutic effect of edaravone is claimed to be associated with the reduction of oxidative stress by eliminating free radicals. HDAC6 inhibitors exert their effect on a different mechanism as they increase the acetylation level of α-tubulin, the building blocks of microtubules and subsequently axonal transport. As a consequence, these inhibitors could eventually serve as a potential new drug candidate and the efficacy in patients is not yet proven. In conclusion, it is clear that a treatment of ALS with a cocktail of drugs which target different pathological pathways could give the best overall therapeutic effect (**Figure 2**).

**Figure 2:**
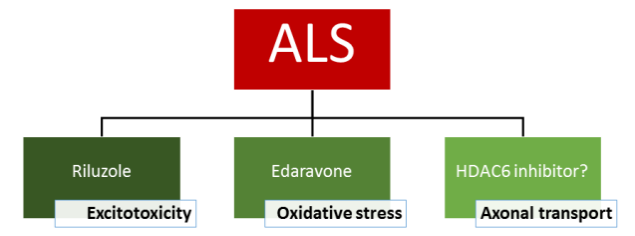
FIGURE 2: Treatment options for ALS.

